# The pseudokinase SgK223 promotes invasion of pancreatic ductal epithelial cells through JAK1/Stat3 signaling

**DOI:** 10.1186/s12943-015-0412-3

**Published:** 2015-07-29

**Authors:** Carole M. Tactacan, Yu Wei Phua, Ling Liu, Luxi Zhang, Emily S. Humphrey, Mark Cowley, Mark Pinese, Andrew V. Biankin, Roger J. Daly

**Affiliations:** Cancer Research Division, The Kinghorn Cancer Centre, Garvan Institute of Medical Research, 384 Victoria St, Darlinghurst, Sydney, NSW 2010 Australia; Department of Biochemistry and Molecular Biology, Monash University, Clayton, VIC 3800 Australia; Wolfson Wohl Cancer Research Centre, Institute of Cancer Sciences, University of Glasgow, Scotland, G61 1BD UK; Department of Biochemistry and Molecular Biology, School of Biomedical Sciences, Monash University, Level 1, Building 77, 23 Innovation Walk, Monash, VIC 3800 Australia

**Keywords:** Pragmin, SgK269, PEAK1, Tyrosine kinase, Pancreatic cancer

## Abstract

**Background:**

Characterization of molecular mechanisms underpinning development of pancreatic ductal adenocarcinoma (PDAC) may lead to the identification of novel therapeutic targets and biomarkers. SgK223, also known as Pragmin, is a pseudokinase and scaffolding protein closely related to SgK269/PEAK1. Both proteins are implicated in oncogenic tyrosine kinase signaling, but their mechanisms and function remain poorly characterized.

**Methods:**

Expression of SgK223 in PDAC and PDAC cell lines was characterized using gene expression microarrays, mass spectrometry (MS)-based phosphoproteomics and Western blotting. SgK223 was overexpressed in human pancreatic ductal epithelial (HPDE) cells via retroviral transduction, and knocked down in PDAC cells using siRNA. Cell proliferation was determined using a colorimetric cell viability assay, and cell migration and invasion using transwells. Expression of markers of epithelial-mesenchyme transition (EMT) was assayed by quantitative PCR. SgK223 and Stat3 signaling was interrogated by immunoprecipitation, Western blot and gene reporter assays. The functional role of specific kinases and Stat3 was determined using selective small molecule inhibitors.

**Results:**

Elevated site-selective tyrosine phosphorylation of SgK223 was identified in subsets of PDAC cell lines, and increased expression of SgK223 detected in several PDAC cell lines compared to human pancreatic ductal epithelial (HPDE) cells and in PDACs compared to normal pancreas. Expression of SgK223 in HPDE cells at levels comparable to those in PDAC did not alter cell proliferation but led to a more elongated morphology, enhanced migration and invasion and induced gene expression changes characteristic of a partial EMT. While SgK223 overexpression did not affect activation of Erk or Akt, it led to increased Stat3 Tyr705 phosphorylation and Stat3 transcriptional activity, and SgK223 and Stat3 associated *in vivo*. SgK223-overexpressing cells exhibited increased JAK1 activation, and use of selective inhibitors determined that the increased Stat3 signaling driven by SgK223 was JAK-dependent. Pharmacological inhibition of Stat3 revealed that Stat3 activation was required for the enhanced motility and invasion of SgK223-overexpressing cells.

**Conclusions:**

Increased expression of SgK223 occurs in PDAC, and overexpression of SgK223 in pancreatic ductal epithelial cells promotes acquisition of a migratory and invasive phenotype through enhanced JAK1/Stat3 signaling. This represents the first association of SgK223 with a particular human cancer, and links SgK223 with a major signaling pathway strongly implicated in PDAC progression.

## Background

Pancreatic ductal adenocarcinoma (PDAC) remains one of most aggressive and lethal of human cancers. The overall 5-year survival rate is <5 %, a statistic that has not changed in almost 50 years [1]. Until recently, gemcitabine was the current standard for post-operative chemotherapy, delaying the development of recurrent disease in some PDAC patients, with a modest improvement in overall survival achieved upon combination with the epidermal growth factor receptor (EGFR) -directed tyrosine kinase inhibitor erlotinib [2]. However, recent studies indicate that the addition of nab-paclitaxel to gemcitabine can provide a significant survival benefit [2], and the combined chemotherapeutic modality Folfirinox has emerged as a more effective treatment than gemcitabine, although at the cost of significant toxicity [2]. Further characterization of the molecular pathways regulating PDAC development and progression may lead to the identification of improved therapeutic strategies, as well as biomarkers that help stratify patients for optimal treatment.

Multiple lines of evidence support a role for deregulated tyrosine kinase signaling in PDAC development and progression. For example, activating KRas mutations represent the earliest known genetic alteration in PDAC [3], and evidence from genetically-modified mouse models support their functional role in this malignancy [4]. Signaling by the EGFR is required for acinar-ductal metaplasia (ADM), an early step in PDAC progression, as well as survival of cells in established ADM and pancreatic intraepithelial neoplasia lesions [5], and overexpression of the EGFR [6] and the related receptor ERBB3 [7] has been detected in PDAC, with aberrant expression being associated with poor prognosis. Alterations can also occur in downstream signaling components. For example, AKT2 gene amplification [8], and loss of the tumour suppressor PTEN [9], occur in this disease. Furthermore, tyrosine phosphorylation of the transcriptional regulator and downstream target of JAK signaling, Stat3, is enhanced in PDAC compared to normal tissue [10], and is associated with poor outcome in resected disease [11]. Importantly, conditional ablation of Stat3 in KRas-driven mouse models of PDAC have confirmed the importance of Stat3 signaling in ADM [12], pancreatic intraepithelial neoplasia (panIN) formation [12, 13] and panIN progression and PDAC development [12, 14].

SgK223/Pragmin [15], and SgK269/PEAK1 [16] are large, closely-related proteins that possess a kinase-like domain at their C-termini. However, these kinase-like domains contain substitutions in key motifs characteristic of *bona fide* kinases, such as the DFG motif responsible for Mg^2+^-ATP binding, where the aspartate residue is substituted by asparagine. Since both proteins lack nucleotide binding activity based on a thermal shift assay, they likely represent pseudokinases [17]. N-terminal to the pseudokinase domain, both proteins contain tyrosine phosphorylation sites that recruit specific SH2 and PTB domain-containing effectors, indicating that SgK223 and SgK269 undertake a scaffolding role during tyrosine kinase signaling. For example, SgK223 binds to Csk, a negative regulator of Src, via SgK223 Y411 [18], while SgK269 binds to Grb2 and Shc1 via Y635 and Y1188 to promote proliferative and morphogenic signals, respectively [19, 20]. Recent work has determined that SgK269 plays a key role during growth factor receptor signaling, mediating a qualitative switch in EGFR output from proliferative/survival signaling to promotion of cell migration/invasion [20].

Importantly, SgK223 and SgK269 both exhibit emerging oncogenic roles. For example, SgK223 promotes cell invasion in colon carcinoma cells exhibiting high Src activity [21], while overexpression of SgK269 promotes growth and aberrant morphogenesis of MCF-10A mammary epithelial cells, and is required for epithelial-to-mesenchymal transition (EMT) and anchorage-independent growth of basal breast cancer cells [19]. In addition, SgK269 is required for efficient tumour formation and metastasis in an orthotopic pancreatic cancer xenograft model [22]. SgK269 is overexpressed in colon, pancreatic and breast cancers relative to normal tissue [19, 22, 16], but the expression profile of SgK223 in human malignancies is poorly characterized.

In this study we demonstrate that SgK223 exhibits enhanced phosphorylation and/or expression in PDAC cell lines and tumours relative to normal controls. In addition, we identify a novel pathway linking SgK223, Stat3 and an invasive phenotype during PDAC development. Overall this work provides important new insights into the signaling and oncogenic function of this pseudokinase scaffold.

## Results

### SgK223 is overexpressed in pancreatic cancer

**Fig. 1:**
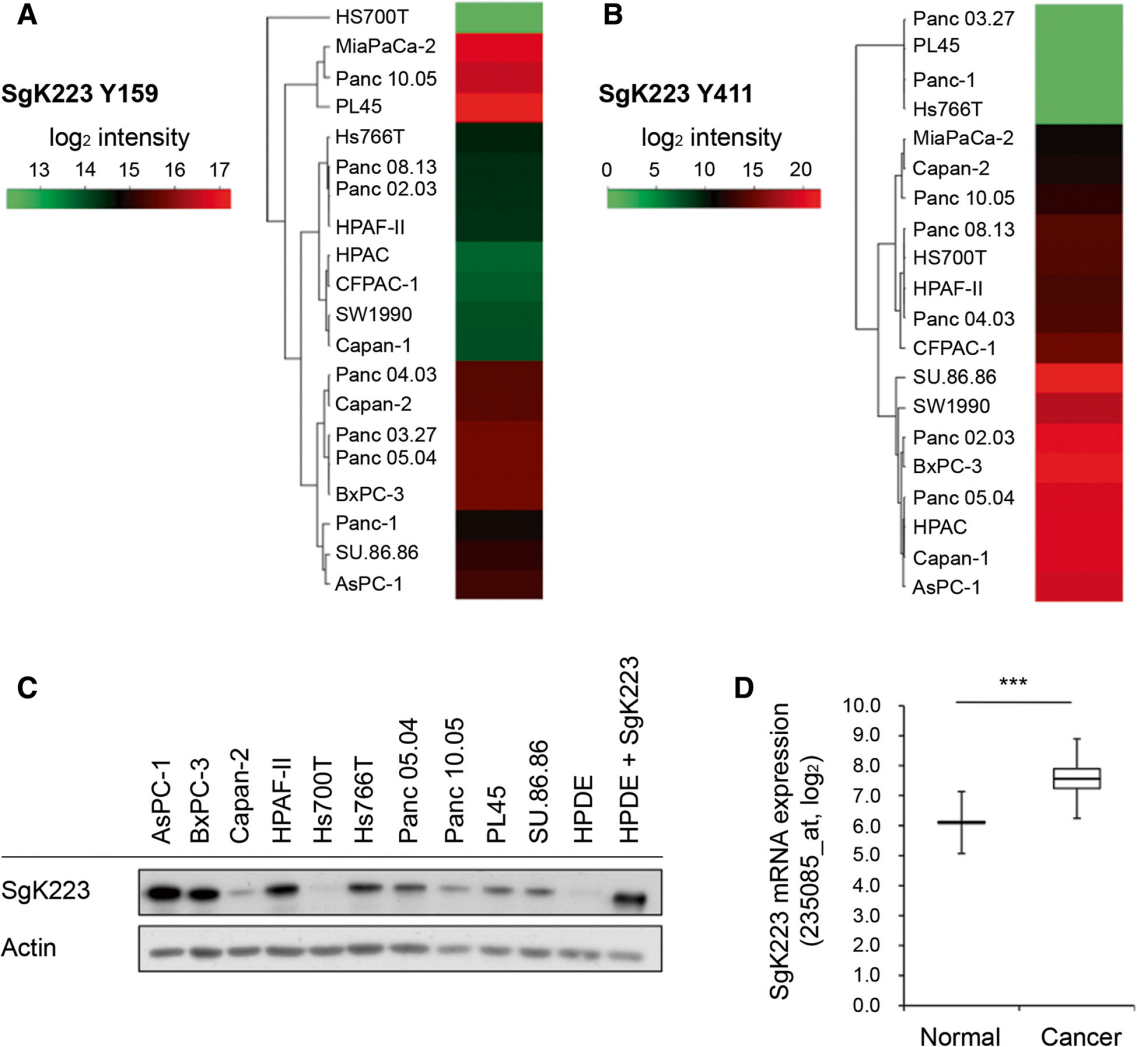
SgK223 in pancreatic cancer. **a**-**b** Site-selective tyrosine phosphorylation of SgK223 across a PDAC cell line panel, determined through mass spectrometry (Humphrey *et al.* manuscript in preparation). The heatmaps indicate relative tyrosine phosphorylation of SgK223 at (**a**) Y159; and (**b**) Y411. **c** SgK223 protein expression across a panel of PDAC cell lines, determined by Western blotting. SgK223 protein expression in the non-transformed pancreatic cell line, HPDE, and an overexpression cell line model utilising HPDE cells are also shown in the same immunoblot. **d** SgK223 is overexpressed in PDAC compared to normal pancreas. The box plot indicates SgK223 mRNA expression in PDAC versus normal pancreatic tissue from the same patient (6 cancer specimens and 6 normal controls were analysed). Boxes represent the interquartile range (IQR) between the 75th percentile and the 25th percentile (Q3 - Q1), and the 50th percentile (median; Q2) is represented as the line within the boxes. Error bars were calculated as Q1 - 1.5 (IQR) and Q3 + 1.5 (IQR). ***indicates significance with a *P*-value < 0.0005

Mass spectrometry-based phosphoproteomic profiling across a wide PDAC cell line panel detected differential phosphorylation of SgK223 Y159 and Y411, suggesting that SgK223 signaling is perturbed in this malignancy (Fig. 1a, b) (Humphrey et al. manuscript in preparation). Three cell lines (MiaPaca2, Panc10.05 and PL45) exhibited relatively high and low levels of tyrosine phosphorylated Y159 and Y411, respectively, while a larger subgroup of 8 cell lines were characterized by increased levels of phosphorylated Y411. These findings led us to assay total SgK223 expression across this panel, and compare this with non-transformed human pancreatic ductal epithelial (HPDE) cells. Western blotting, using a custom rabbit polyclonal antibody, revealed that SgK223 was overexpressed relative to HPDE cells in all pancreatic cancer cell lines tested except Hs700T (Fig. 1c). Of particular note was the overexpression of SgK223 in the cell lines AsPC-1 and BxPC-3, members of the cell line subgroup characterized by high levels of Y411 (Fig. 1c). In order to determine whether SgK223 is overexpressed in primary PDAC, we analysed our own gene expression data for PDAC specimens and macro- and microscopically normal-appearing pancreatic tissue from the same patients [23]. This revealed a marked increase in SgK223 expression in PDAC versus normal controls (Fig. 1d). Thus data from cell line models and human tissue specimens are suggestive of a role for SgK223 in PDAC development and/or progression.

### Overexpressing SgK223 in HPDE cells increases cell migration and invasion

In order to characterize the functional role of SgK223 in pancreatic cancer, we overexpressed this protein in HPDE cells via retroviral infection, using a construct encoding rat SgK223 [15]. This led to an expression level comparable to that of AsPC-1 and BxPC-3 pancreatic cancer cells (Fig. 1c). Phosphoproteomic profiling was undertaken to characterize site-selective phosphorylation of the ectopically-expressed SgK223, using BxPC-3 PDAC cells as a positive control. Tyrosine phosphorylation of endogenous SgK223 at Y411 was detected in HPDE and HPDE/SgK223 cells at a comparable level, and at approximately 4-fold higher levels in BxPC-3 cells. In addition, compared with the phosphorylation of endogenous Y411 in HPDEs, a slightly higher level of tyrosine phosphorylation of Y391, the rat equivalent of Y411, was detected in the HPDE/SgK223 cells. Phosphorylation of rat Y146, equivalent to human Y159, was not detected (data not shown). Therefore, in the context of SgK223 phosphorylation, HPDE/SgK223 cells most closely model the subset of PDAC lines with high phosphorylation of Y411 and low/undetectable phosphorylation of Y159 (eg Capan-1, SW1990, Fig. 1a, b).

**Fig. 2:**
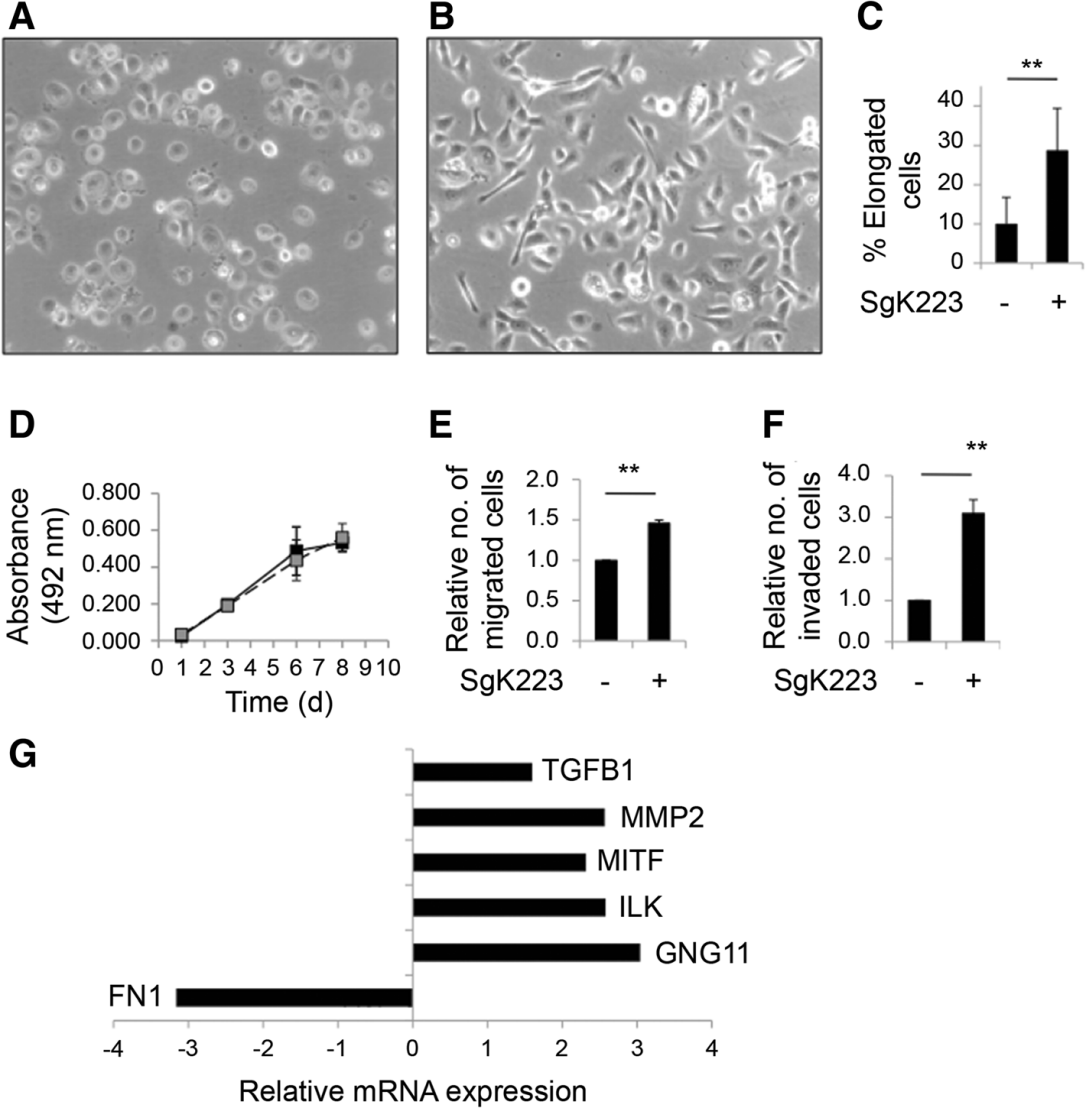
SgK223 overexpression in HPDE cells enhances cell migration and invasion. SgK223 alters the morphology of HPDE cells grown in monolayer. Light microscope images of HPDE cells containing either: (**a**) the vector-control; or (**b**) the SgK223-expression construct. **c** The relative percentage of elongated cells (cells with lengths double their width) in the vector-control and the SgK223-overexpression cell lines. Data represent mean and the standard error of mean (SEM). **indicates significance with a *P*-value < 0.005. **d** SgK223 does not affect the proliferation rates of HPDE cells grown in monolayer. The MTS assay was used to quantify the number of cells, over time. Graph shows the relative proliferation rates between the vector-control (black) and SgK223-overexpressing (grey) HPDE, cell lines. Data points represent mean and SEM at different time points. (E) Relative cell migration. Data are expressed relative to the vector control, which is arbitrarily set at 1.0. Bars represent SEM of three independent experiments, **indicates significance with a *P*-value < 0.005. **f** Relative cell invasion, data are represented as in (**e**). **g** Expression analysis on EMT-associated genes. The histogram indicates relative fold-difference in transcript levels of 6 genes with significant changes in expression in the SgK223-overexpressing, relative to the vector-control, cell line

SgK223 overexpression altered the morphology of HPDE cells, resulting in an elongated and more refractile mesenchymal-like phenotype (Fig. 2a–c), but did not affect anchorage-dependent cellular proliferation (Fig. 2d). To determine whether SgK223 contributed to a motile and invasive phenotype, transwell assays were undertaken. SgK223 overexpression significantly increased cellular migration (Fig. 2e), and to a greater extent, enhanced cellular invasion (Fig. 2f). Since acquisition of a mesenchymal morphology and increased motility and invasiveness are suggestive of epithelial-to-mesenchymal transition (EMT), we characterized the expression of 84 EMT-related genes using a BD Bioscience RT^2^ Profiler™ PCR Array. Of the 21 genes on the array that are normally downregulated during EMT, none showed significant change. Of the 63 genes on the array that are normally upregulated during EMT, 6 showed a significant change, and the majority of these (5/6) increased in expression (Fig. 2g). The latter included TGFB1 and MMP2. These findings indicate that SgK223 overexpression induces a partial EMT.

### The impact of SgK223 on downstream signaling pathways

**Fig. 3:**
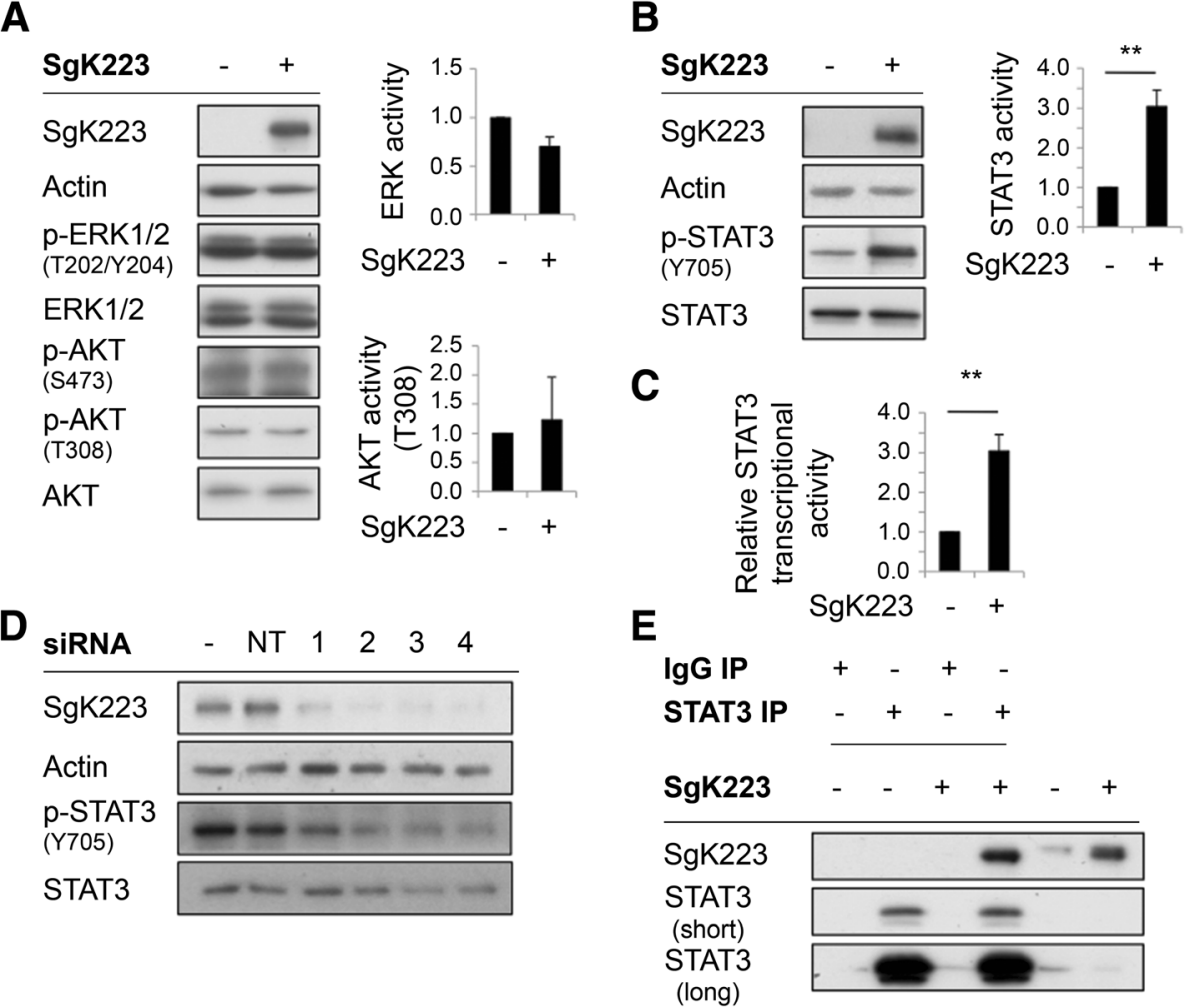
The effect of SgK223 overexpression on specific signaling pathways. **a** SgK223 does not affect activation of Erk or Akt. Western blots were undertaken as indicated. Phospho-Erk and -Akt (T308) signals were quantitated by densitometry and normalized for total expression, and expressed relative to the value for control cells, which was arbitrarily set at 1.0. The histograms represent the mean +/− the SE from three independent experiments. **b** SgK223 enhances Stat3 tyrosine phosphorylation (pY705). Data are normalized and expressed as in the previous panel. **indicates significance with a *P*-value < 0.005. **c** SgK223 enhances Stat3 transcriptional activity. Data are expressed relative to the value for control cells, which was arbitrarily set at 1.0. The histograms represent the mean +/− the SE from three independent experiments. ** indicates significance with a *P*-value < 0.005. **d** SgK223 knockdown in AsPC-1 PDAC cells decreases Stat3 activation. Two days following transfection using non-targeting (NT) or individual siRNAs directed against SgK223, cell lysates were prepared and Western blotted as indicated. **e** SgK223 and Stat3 associate *in vivo*. Control (−) or SgK223-overexpressing (+) HPDE cells were subject to immunoprecipitation (IP) using Stat3 antibodies, or corresponding mouse IgG (negative control). IPs or total cell lysates before IP were Western blotted as indicated. The upper and lower panel of the Stat3 blot represent a short and long exposure, respectively

Next, we sought to determine how SgK223 elicits these biological effects by characterizing its impact on known downstream signaling pathways. While the related SgK269 modulates signaling via the focal adhesion proteins p130Cas and paxillin [16], phosphorylation of p130Cas (on Y249) and paxillin (on Y31) was similar in control and SgK223-overexpressing cells (data not shown). In addition, activation of Erk and Akt was similar in control and SgK223-overexpressing cells (Fig. 3a). However, Stat3 tyrosine phosphorylation was enhanced approximately 3-fold (Fig. 3b). In order to determine whether this was accompanied by an increase in Stat3 transcriptional activity, we undertook reporter assays (Fig. 3c). Indeed, a corresponding increase in expression from a Stat3 reporter construct was observed in SgK223-overexpressing cells. Additionally, knocking down SgK223 in AsPC-1 cells using several independent siRNAs decreased Stat3 tyrosine phosphorylation (Fig. 3d), demonstrating that endogenous high levels of SgK223 in PDAC cells promote Stat3 activation. Since SgK223 has a known scaffolding role in signal transduction [18], we determined whether SgK223 and Stat3 associate. Indeed, SgK223 co-immunoprecipitated with Stat3 (Fig. 3e).

### SgK223 signals via JAK1 to enhance STAT3 activation

**Fig. 4:**
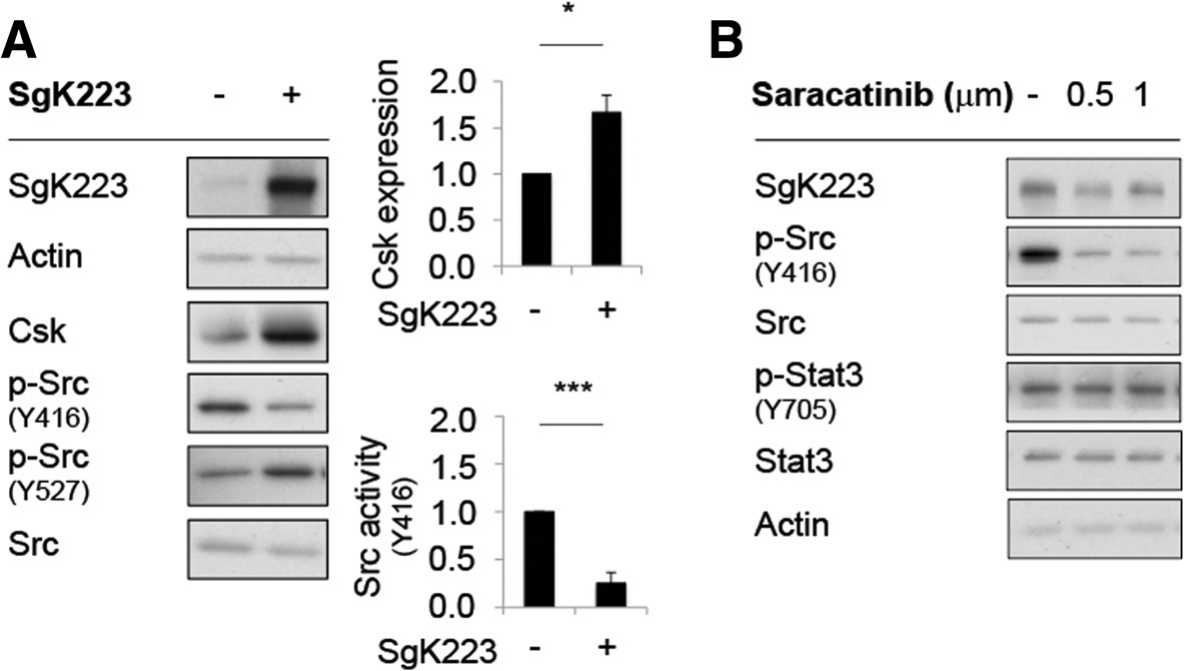
Enhanced Stat3 activation in SgK223-overexpressing cells is not mediated via Src. **a** Overexpression of SgK223 in HPDE cells increases Csk expression and decreases Src activity. Data are normalized and expressed as in the previous figures. *indicates significance with a *P*-value < 0.05, ***indicates significance with a *P*-value < 0.0005. **b** The Src inhibitor Saracatinib does not affect Stat3 expression or activation

Next, we set out to determine how SgK223 enhances Stat3 signaling. Known Stat3 kinases include Src family kinases, the EGFR, and the JAKs. While total levels of Src were similar in the SgK223-overexpressing and vector control cells (Fig. 4a), phosphorylation of Src Y416 (Y419 in human Src) on the activation loop was significantly lower in the former cell type, while phosphorylation on the negative regulatory site Y527 (Y530 in human Src) was enhanced. Consistent with these observations, expression of Csk, which phosphorylates Src on Y530, was significantly higher upon SgK223 overexpression (Fig. 4a). In addition, treatment of HPDE cells overexpressing SgK223 with the selective Src inhibitor saracatinib did not affect Stat3 phosphorylation (Fig. 4b). These data rule out SFKs as the mediators of enhanced Stat3 phosphorylation in SgK223-overexpressing cells.

**Fig. 5:**
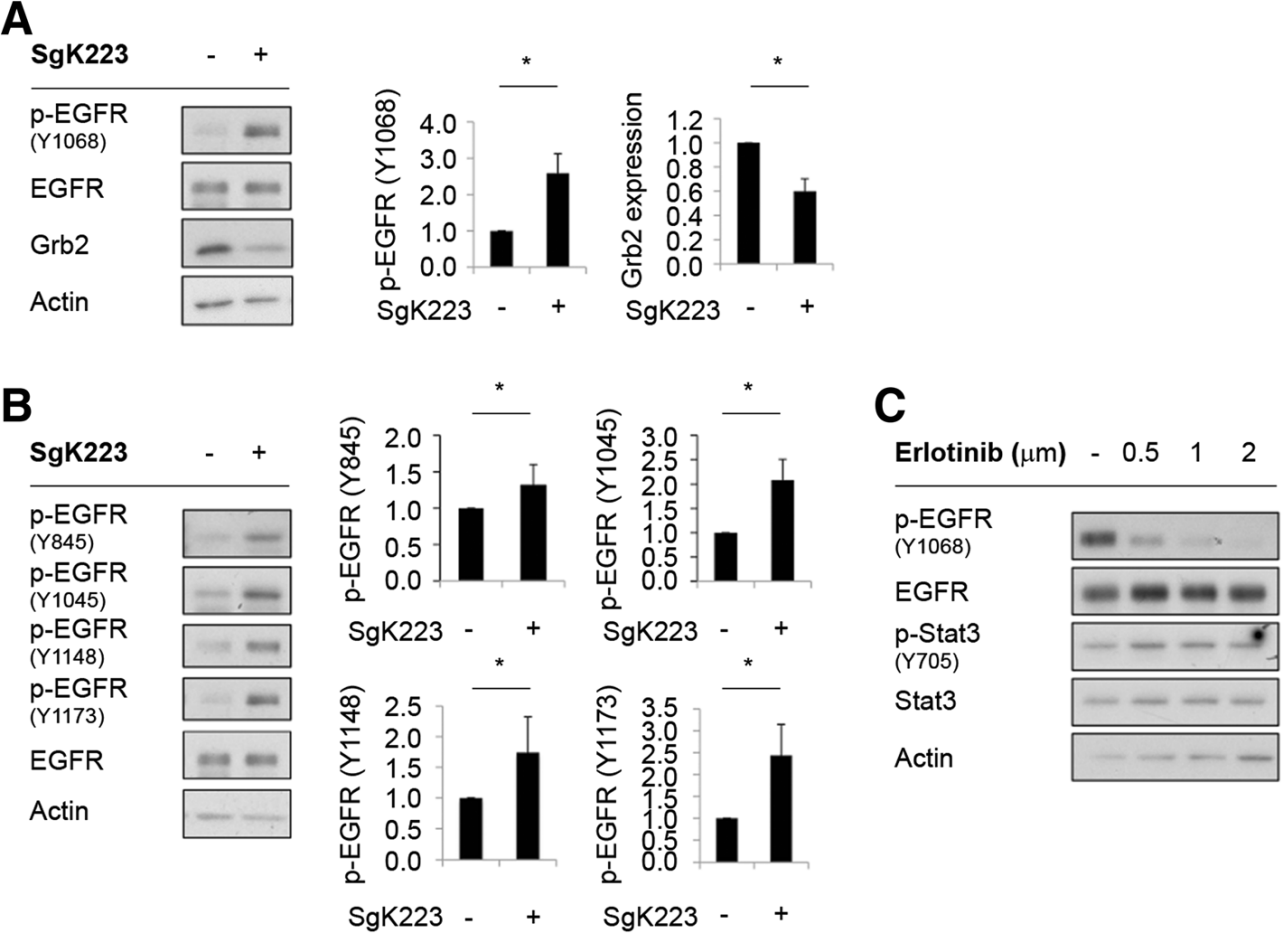
SgK223 and EGFR signaling. **a** Effect of SgK223 overexpression on EGFR Y1068 phosphorylation and Grb2 expression. **b** Effect of SgK223 overexpression on additional EGFR tyrosine phosphorylation sites. Data are normalized and expressed as in the previous figs. **c** The EGFR inhibitor, erlotinib, does not affect Stat3 phosphorylation in SgK223-overexpressing cells

With regard to EGFR signaling, SgK223 overexpression increased phosphorylation of the EGFR on Y1068 and decreased Grb2 levels (Fig. 5a). These data were of interest because Y1068 represents a binding site for both Stat3 and Grb2 [24], so these changes may lead to enhanced EGFR signaling through Stat3. Furthermore, SgK223 also increased phosphorylation of the EGFR on Y845, Y1045, Y1148 and Y1173 (Fig. 5b). However, treatment of the SgK223-overexpressing cells with the selective EGFR tyrosine kinase inhibitor erlotinib did not affect Stat3 activation, despite clear inhibition of EGFR Y1068 phosphorylation (Fig. 5c), indicating that the enhanced Stat3 tyrosine phosphorylation in SgK223-overexpressing cells was not mediated by the EGFR.

**Fig. 6:**
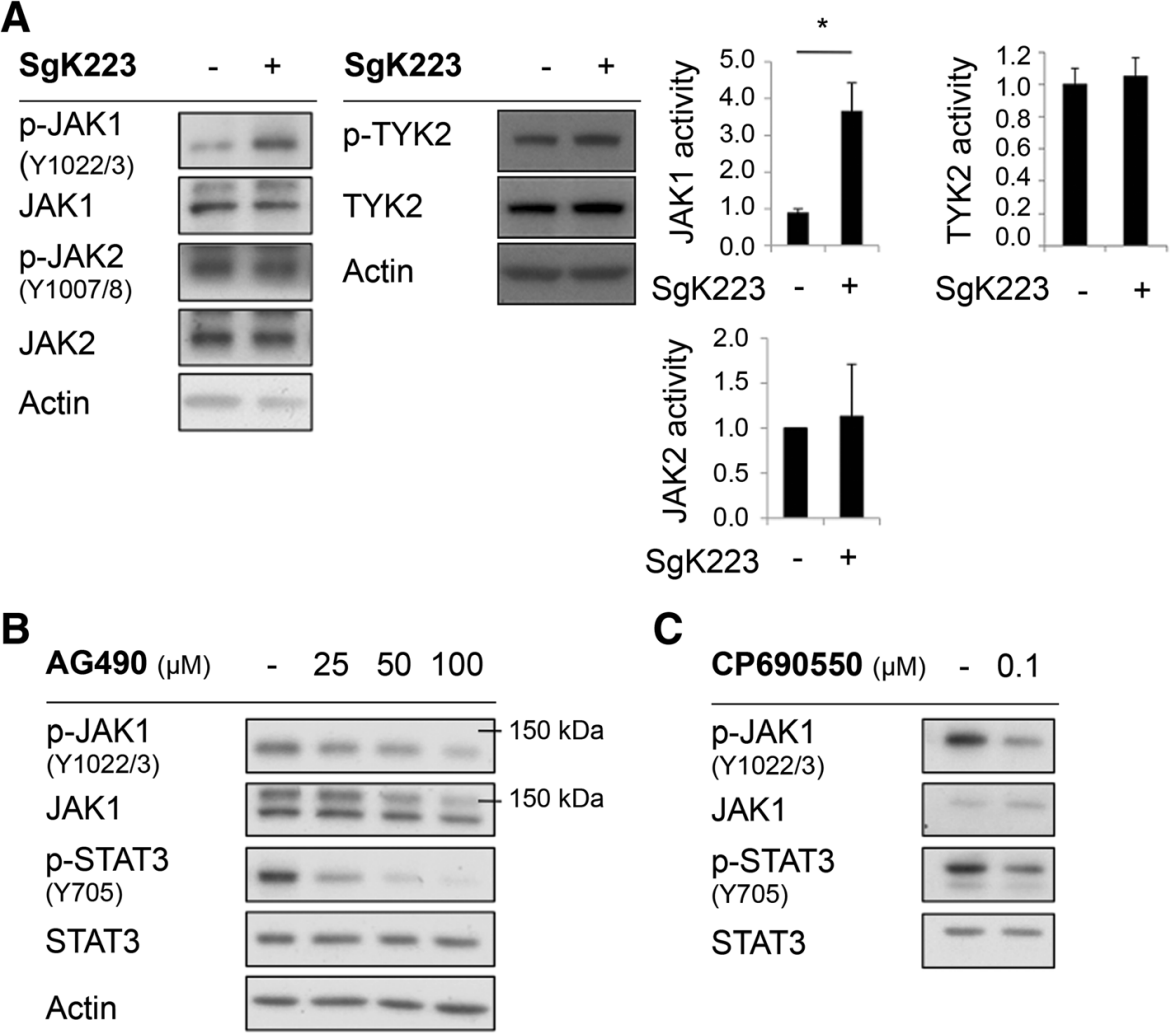
SgK223 signals via JAK1/Stat3. **a** Overexpression of SgK223 enhances JAK1 activation. Data are normalized and expressed as in Fig. 3. *indicates significance with a *P*-value < 0.05. **b**-**c** Activation of Stat3 in SgK223-overexpressing cells is JAK-dependent. **b** AG490 or **c** CP690550 were applied for 2 h prior to cell lysis. Cell lysates were then Western blotted as indicated

Next, we characterized the role of the JAKs. Western blotting with appropriate phosphospecific antibodies revealed that JAK1 activity was significantly enhanced upon SgK223 overexpression, while JAK2 and TYK2 activities remained unchanged (Fig. 6a). In addition, treatment of the SgK223-overexpressing cells with the selective JAK inhibitor AG490 led to a dose-dependent reduction of JAK1 and Stat3 phosphorylation, identifying JAK1 as the likely upstream Stat3 kinase in these cells (Fig. 6b). The role of the JAKs in Stat3 phosphorylation was confirmed using the different selective JAK inhibitor CP690550 (Fig. 6c). Of note, although we were able to co-immunoprecipitate Stat3 with SgK223 (Fig. 3e), we could not detect association of JAK1 with SgK223 via this approach.

Since signaling by IL-6 via the common signal transducing component of the IL6 cytokine receptor family, gp130, represents a key mechanism for activation of JAK/Stat signaling, we assayed the status of this pathway in our model system. A commercial antibody array was used to assay the levels of 36 different cytokines in cell culture supernatants derived from control or SgK223-overexpressing cells. While IL-6 could not be detected in supernatants from either cell pool, expression of several other cytokines, including G-CSF and IL-1α, was detectable but not altered upon SgK223 overexpression (data not shown). In addition, expression of gp130, as determined by Western blotting, was similar in the two cell pools (data not shown). Therefore, enhanced JAK1 activation upon SgK223 overexpression does not reflect increased IL-6 production or gp130 expression.

### SgK223 enhances cell invasion through STAT3 signaling

**Fig. 7:**
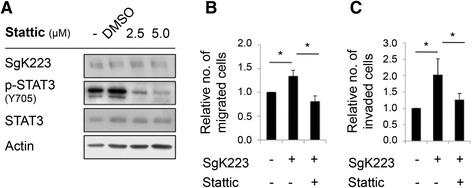
Promotion of cell migration and invasion by SgK223 is Stat3-dependent. **a** Inhibition of Stat3 activation using STATTIC. SgK223-overexpressing HPDE cells were treated with Stat3 inhibitor, STATTIC, at the indicated concentrations for 2 h prior to cell lysis. Cell lysates were then Western blotted as indicated. **b**-**c** Effect of STATTIC on cell migration and invasion. Treatment of SgK223-overexpressing cells with STATTIC (2.5 μM) reduced migration (**b**) and invasion (**c**) to levels comparable to that of untreated control cells. Cell migration and invasion are expressed relative to the value for untreated control HPDE cells, which is arbitrarily set at 1.0. Data represent the mean +/− SEM of three independent experiments. *indicates significance with a *P*-value < 0.05

To determine the functional role of Stat3 downstream of SgK223, we utilized the small molecule Stat3 inhibitor STATTIC [25]. Treatment of the SgK223-overexpressing cells with STATTIC markedly reduced Stat3 phosphorylation (Fig. 7a) as well as cell migration (Fig. 7b) and invasion (Fig. 7c). Thus Stat3 activation is required for the enhanced migratory and invasive phenotype of SgK223-overexpressing pancreatic ductal epithelial cells.

## Discussion

Several studies have implicated Stat3 activation in PDAC development and progression [12, 11, 13, 14, 10]. Previous reports indicated that the major signaling pathway leading to activation of Stat3 in PDAC is cytokine-induced tyrosine phosphorylation of the signaling co-receptor gp130 and JAK activation [12], and this can occur in a cell autonomous fashion [12, 13], as well as via a cell non-autonomous mechanism involving IL-6 production by infiltrating myeloid cells [14]. Increased production and/or exposure to cytokines, and upregulation of gp130, represent identified mechanisms for the enhanced activation of Stat3 detected in PDAC versus normal tissue [12–14]. Our identification of increased expression of SgK223 in PDAC versus normal tissue, and demonstration that SgK223 is sufficient to increase Stat3 activation in HPDE cells and is required for Stat3 phosphorylation in AsPC-1 PDAC cells, now identify aberrant expression of this pseudokinase as an additional mechanism for enhancement of Stat3 signaling in PDAC. While all details of SgK223 action on this pathway have yet to be resolved, our work identifies certain key aspects. First, since JAK1 activation was enhanced in SgK223-overexpressing cells, and selective JAK inhibitors could normalize Stat3 phosphorylation, SgK223 must act upstream of JAK1 to amplify Stat3 signaling. Second, increased production of IL6, and altered expression of gp130, do not appear to be involved. Third, since SgK223 associates with Stat3, this implicates the scaffolding function of SgK223 in regulation of Stat3 phosphorylation, although SgK223 does not itself ‘bridge’ Stat3 and JAK1. The possibility that SgK223 complexes both gp130 and Stat3, or alters the activity of particular protein tyrosine phosphatases, such as TC-PTP [26], towards Stat3, is currently under investigation. Whatever the mechanism, the phosphorylated Stat3 generated in SgK223-overexpressing cells is competent for nuclear translocation and transcriptional activation of target genes, as confirmed by our reporter assays.

Reflecting their relationship as paralogues, SgK223 and SgK269 exhibit similarities and differences in function and signal output. Both proteins regulate cell morphology, promoting a more elongated phenotype [19, 18] and a partial EMT [19]. With regard to the latter process, it is noteworthy that increased expression of either protein led to enhanced expression of MITF, a transcription factor that regulates the balance between migration/invasion and proliferation in melanoma cells, and particular TGFβ family members, that are known to promote acquisition of a mesenchymal phenotype (Fig. 2) [19, 27, 28]. In addition, both SgK223 and SgK269 modulate the tyrosine phosphorylation of specific erbB receptors. The Klemke group [22] demonstrated that SgK269 associated with erbB2, and SgK269 knockdown decreased phosphorylation of this receptor on specific sites, while we observed enhanced EGFR tyrosine phosphorylation upon SgK223 overexpression. However, while SgK269 enhanced site-selective tyrosine phosphorylation of paxillin and p130Cas [16], this direct impact on focal adhesion signaling was not observed in our SgK223 overexpression model. In addition, while SgK269 increases Erk activation [19, 16], this was not observed in HPDE cells overexpressing SgK223 (Fig. 3). This likely reflects the absence of a direct Grb2 binding site in SgK223, while SgK269 recruits this adaptor via Y635 [19].

Interestingly, previous studies reported positive regulation of Src by SgK223 and SgK269 [22, 18]. In the case of the former pseudokinase, this reflected sequestration of Csk in the cytosol by SgK223, leading to activation of plasma membrane-localized Src [18]. However, this was demonstrated using transient expression assays in gastric epithelial cells, and in our HPDE cells stably expressing SgK223, Src activity was decreased, likely reflecting a robust increase in Csk expression in the SgK223-overexpressing cells, which may represent a negative feedback mechanism. Taking these data and those from the Hatakeyama group in combination, the effect of SgK223 on Src may be context-dependent, and vary according to the relative abundance of Src and its regulators in specific subcellular localizations.

An additional conserved feature of SgK223 and SgK269 is their ability to enhance activation of Stat3 ([19], this paper). In the case of SgK223, we utilized the Stat3 inhibitor Stattic to demonstrate that a key role for SgK223-induced Stat3 signaling is enhanced cellular migration and invasion. The cellular function of Stat3 signaling downstream of SgK269 has yet to be defined, although mutation of Y635 to phenylalanine, which abrogates SgK269-mediated activation of both Erk and Stat3, negatively impacts upon both proliferation in 3D culture and cellular invasion [19]. Consequently, it appears likely that Stat3 represents a common effector utilized by both pseudokinases to drive acquisition of a migratory and invasive phenotype. This highlights an important oncogenic role for both SgK223 and SgK269 in PDAC, given that both are overexpressed in this malignancy (this paper, [22]) and the critical role played by Stat3 in PDAC development [12–14] and metastasis [29]. In addition, the strong links between these pseudokinases and Stat3 signaling indicate that SgK223 and SgK269 represent candidate biomarkers for responsiveness to targeted therapies directed at the JAK/Stat3 pathway.

## Conclusions

Accumulating evidence indicates that pseudokinases play key roles in regulating intracellular signaling pathways by functioning as allosteric regulators or scaffolds [30, 20]. The signaling potential of pseudokinases is also reflected in the growing number of these proteins implicated in human disease, including cancer [31]. In this manuscript, we extend these findings by demonstrating a novel role for the pseudokinase SgK223 in PDAC. Specifically, SgK223 is overexpressed in PDAC relative to normal pancreatic tissue and promotes acquisition of a migratory and invasive phenotype in pancreatic ductal epithelial cells through enhanced JAK1/Stat3 signaling. This represents the first association of SgK223 with a particular human cancer, and links SgK223 with a major signaling pathway strongly implicated in PDAC progression.

## Materials and methods

### Plasmids

The retroviral expression vector pBabe-puro/myc-Pragmin was a kind gift from Serge Roche, Centre for Biochemical and Macromolecular Research (CRBM), Montpellier Cedex, France [21], and was previously generated in the laboratory led by Manabu Negishi, Kyoto University, Kyoto, Japan [15].

### Cell culture and generation of stable cell lines

HPDE cells stably expressing the murine ecotropic receptor were maintained in Keratinocyte Serum-Free Media (K-SFM), supplemented with 2.5 μg human recombinant EGF and 25 mg bovine pituitary extract per litre (Gibco®, Life Technologies, USA). All pancreatic cancer cell lines were obtained from the American Type Culture Collection (ATCC, USA) and were maintained according to ATCC guidelines. The packaging cell line PlatE was used for retrovirus production, and HPDE cells were infected with retrovirus using the method previously described [32]. Puromycin (Sigma-Aldrich, USA) was used to select for pBabe-puro-positive HPDE cells at a concentration of 200 ng/ml for 14 days.

### Tyrosine phosphorylation profiling

Global tyrosine phosphorylation profiling was undertaken by immunoaffinity purification and liquid chromatography-tandem mass spectrometry, as previously described [33, 34], using heavy amino acid-labelled synthetic phosphotyrosine-containing peptides for data normalization [35].

### Antibodies, inhibitors and siRNA treatment

All antibodies were from Cell Signaling Technology, except Actin (Santa Cruz, USA) and SgK223. The affinity-purified rabbit antibody selective for SgK223 was generated by Cambridge Research Biochemicals Ltd. by standard procedures. [C]-PTDHSNSTTWHRLHPTDGS-amide was used as the antigen. The following inhibitors were used in this study: Erlotinib hydrochloride (Symansis, USA); AG490, CP690550 (Tocris Bioscience, USA); AZD0530 (saracatinib) (a kind gift from Ian Steet, Cancer Therapeutics CRC, Vic, Australia); and STAT3 Inhibitor V, STATTIC (Calbiochem, Merck Millipore, Germany). Cells were treated with inhibitors for 2 h at the indicated concentrations prior to cell lysis for immunoblot analysis. The Universal Negative Control #1 (SIC001) and the SgK223-selective siRNAs (SASI_Hs02_0031603-3 to −6) were obtained from Sigma-Aldrich. siRNA was applied to cells (final concentration, 5 nM) using Lipofectamine 2000 (Invitrogen, USA) transfection reagent using the method previously described [32].

### Immunoprecipitation and immunoblotting

Cell lysates for immunoprecipitation and immunoblotting were prepared using normal lysis buffer [36]. Both Myc-Tag (#2272, Cell Signaling Technology) and SgK223 (Cambridge Research Biochemicals Ltd) antibodies were used to confirm expression from the pBabe-puro/myc-Pragmin plasmid in HPDE cells.

### Cell proliferation, migration and invasion assays

Cell proliferation assays were conducted using MTS reagent as previously described [32]. For migration and invasion assays, HPDE cells were starved for 24 h with K-SFM without supplements, prior to starting the assays. Assays were performed using cell culture inserts with 8 μM pore size PET filters designed for 24-well plates, uncoated (BD Falcon™ Cell Culture Inserts; BD Biosciences) and coated in Matrigel (BD Biocoat™ Growth Factor Reduced MATRIGEL™ Invasion Chamber; BD Biosciences), for assaying migration and invasion, respectively. For invasion assays, the Matrigel was rehydrated with K-SFM without supplements, for 2 h at 37 °C in 5 % CO_2_, prior to cell seeding. The lower compartment of each well was filled with 0.75 ml of K-SFM supplemented with EGF and bovine pituitary extract. The upper compartment, within the inserts, was filled with 0.5 ml of resuspended cell solution (2.5 × 10^4^ cells/0.5 ml of K-SFM without supplements). Cells were subsequently incubated for 24 h at 37 °C in 5 % CO_2_ to allow migration/invasion through the filter, after which, remaining cells in the upper compartment were removed by swabbing with a cotton tip. The filter from the insert was subsequently stained using Diff Quick solution (Lab Aids Pty. Ltd., Australia). Stained cells that had migrated/invaded through to the underside of the filter were subsequently photographed and quantified using ImageJ software (version 10.2).

### Quantitative RT-PCR analysis of EMT genes

The RNeasy Kit (Qiagen) was used to extract RNA and reverse transcription was conducted with the RT2 First Strand Kit (Qiagen). The RT2 Profiler PCR Array for Human EMT (PAHS-0902E-4; Qiagen) was used for gene expression profiling and quantitative RT-PCR was carried out on an Applied Biosystems ABI 7900 qPCR machine. Data were analyzed using the 2^ΔΔCt^ method.

### STAT3 reporter assay

The pGL4.23/APRE-luc construct used in this assay was a kind gift from Matthias Ernst (Walter and Eliza Hall Institute of Medical Research, Melbourne, VIC, Australia). The construct was made by inserting 8 × APRE sites into a firefly luciferase pGL4.23 (Promega, USA) vector, using a method modified from Takeda, T. et al. (1994) [37]. A renilla luciferase pRL-TK (Promega) vector was used as the corresponding co-transfection control. Untransfected cells were used as negative controls, while IL-6-treated cells were used as positive controls. Positive control cells were treated with IL-6 (100 ng/ml) for 6 h prior to the measurement of the assay on day three. On day one, HPDE_pBabe-puro and HPDE_pBabe-puro/myc-Pragmin cells were seeded, in replicate, at 2 × 10^5^ cells/well, in a 24-well plate. On day two, 2 μg of pGL4.23/APRE-luc (firefly) and 0.2 μg of pRL-TK (renilla) vectors were co-transfected into the cells using Lipofectamine 2000 (Invitrogen) according to the transfection reagent manufacturer instructions. On day three, cells were lysed, and the luminescence from firefly and renilla luciferase activity was measured using reagents from the Dual-Luciferase® Reporter Assay System (Promega) and read on a FLUOstar OPTIMA (BMG Labtech, Germany).

### Statistical tests

These were by standard t-tests, unless otherwise indicated.

## Abbreviations

ADM: Acinar-ductal metaplasia
EGFR: Epidermal growth factor receptor
EMT: Epithelial-to-mesenchymal transition
HPDE: Human pancreatic ductal epithelial
IP: Immuneprecipitation
IQR: Interquartile range
panIN: pancreatic intraepithelial neoplasia
PDAC: Pancreatic ductal adenocarcinoma
SE: Standard error
SEM: Standard error of mean
